# Increased risk of severe congenital heart defects in offspring exposed to selective serotonin-reuptake inhibitors in early pregnancy – an epidemiological study using validated EUROCAT data

**DOI:** 10.1186/1471-2393-14-333

**Published:** 2014-09-25

**Authors:** Tanja Majbrit Knudsen, Anne Vinkel Hansen, Ester Garne, Anne-Marie Nybo Andersen

**Affiliations:** Department of Public Health, University of Copenhagen, Oester Farimagsgade 5, 1014 Copenhagen K, Denmark; Paediatric Department, Hospital Lillebaelt, Skovvangen 2-6, 6000 Kolding, Denmark

**Keywords:** SSRI drugs, Antidepressant, Congenital heart defects, Socioeconomic status, EUROCAT, Cohort study

## Abstract

**Background:**

Previous studies suggest a possible association between maternal use of selective serotonin-reuptake inhibitors (SSRIs) during early pregnancy and congenital heart defects (CHD). The purpose of this study was to verify this association by using validated data from the Danish EUROCAT Register, and secondary, to investigate whether the risk differs between various socioeconomic groups.

**Methods:**

We conducted a cohort study based on Danish administrative register data linked with the Danish EUROCAT Register, which includes all CHD diagnosed in live births, fetal deaths and in pregnancies terminated due to congenital anomalies. The study population consisted of all registered pregnancies (n = 72,280) in Funen, Denmark in the period 1995–2008. SSRI-use was assessed using The Danish National Prescription Registry, information on marital status, maternal educational level, income, and country of origin from Statistics Denmark was used as indicators of socioeconomic situation, and the CHD were studied in subgroups defined by EUROCAT. Logistic Regression was used to investigate the association between redeemed prescriptions for SSRIs and CHD.

**Results:**

The risk of severe CHD in the offspring of the 845 pregnant women who used SSRIs during first trimester increased four times (AOR 4.03 (95% CI 1.75-9.26)). We found no increased risk of septal defects. Socioeconomic position did not modify the association between maternal SSRI-use during pregnancy and severe CHD.

**Conclusion:**

This study, which is based on data with high case ascertainment, suggests that maternal use of SSRIs during first trimester increases the risk of severe CHD, but does not support findings from previous studies, based on administrative register data, regarding an increased risk of septal defects. The study was unable to document an interaction between socioeconomic status and maternal SSRI-use on the risk of severe CHD.

## Background

The prevalence of depression is high among women in the childbearing age [1]. Selective serotonin-reuptake inhibitors (SSRIs) are currently the most prescribed drugs for depression and depressive symptoms. Partly, because some pregnancies are unplanned, and abrupt withdrawal of SSRIs is associated with complications, maternal use of SSRIs during pregnancies is common [2–4]. The proportion of pregnant women in Denmark who use SSRIs has increased by a factor eight during the period 1997–2006 [5], and similar increases have been observed for other countries [4, 6, 7]. Several studies support the evidence that users of antidepressant tend to have lower socioeconomic status compared with non-users of SSRIs [8–10]. Serotonin plays a role in the early development of the heart [11]. It is therefore plausible that maternal exposure to SSRIs can result in congenital heart defects (CHD) in the offspring.

Recent epidemiological studies have proposed a possible association between maternal use of SSRIs during early pregnancy and risk of CHD in the offspring. However, results for specific types of SSRIs are conflicting [12–23], and some studies have not been able to detect an association [24–30]. The majority of the studies have based their information about congenital anomalies on administrative register data, for which the validity of these conditions is questionable and often limited to live born children [31]. EUROCAT, a European network for the epidemiologic surveillance of congenital anomalies, has developed common standards for diagnosis and registration of congenital anomalies [32]. The Danish EUROCAT Register includes data on all pregnancy outcomes with congenital anomalies within the Funen region of Denmark, i.e. all congenital anomalies diagnosed in live born children, in fetal deaths and in foetuses from pregnancies that were terminated due to foetal anomaly. All diagnosed are verified and coded by a trained paediatrician [32, 33]. The Danish EUROCAT Register contains high quality data on congenital anomalies, in addition to high case ascertainment [32]. Using data from the EUROCAT Registry provides a good opportunity to verify and qualify previous findings in relation to the association between SSRIs and CHD.

Some studies have shown, that CHD are more frequent in the offspring of women with low socioeconomic position [34–36], which may due to differences in lifestyle factors and life conditions between socioeconomic groups [37]. In a public health perspective is it relevant to investigate whether socioeconomic status, as an indicator for various lifestyle factors and life conditions during pregnancy, interacts with use of SSRIs with respect to the risk of CHD. Differences in risk may reflect different vulnerabilities across socioeconomic groups.

The primary aim of this study was to verify the association between maternal first trimester use of SSRIs and CHD using the high quality anomaly data as provided by EUROCAT (European Surveillance of Congenital Anomalies). We also sought to examine whether maternal socioeconomic status has an influence on the association between SSRI-use and CHD.

## Methods

### Study design and study population

We conducted a cohort study which included all identifiable pregnancies with an estimated last menstrual period (LMP) between 2nd February 1995 and 4th October 2008 in the Funen County, Denmark. The study population covered approximately 9% of all pregnancies in Denmark during the same period of time. In total, 74,381 pregnancies were identified through The Danish Medical Birth Registry (live births and stillbirths) [38] and The Danish National Hospital Register (terminations of pregnancy and miscarriages) [39]. Information on maternal exposure to SSRIs was obtained from The Danish National Prescription Registry [40], while the population registers in Statistics Denmark provided data on measures for maternal socioeconomic status [41, 42]. Data from the administrative registers were individually linked with data from the Danish EUROCAT Register in Statistics Denmark’s server. The Danish EUROCAT Register contains information on all congenital anomalies in live births, fetal deaths with gestational age (GA) from 20 weeks and onwards, and on all terminations of pregnancy after prenatal diagnosis of fetal anomaly (TOPFA). The register covers the County of Funen from 1995 and onwards [32]. We excluded 2,101 observations with missing data on socioeconomic measures from Statistics Denmark. None of these were registered in EUROCAT with congenital anomalies. The STROBE (Strengthening the Reporting of Observational Studies in Epidemiology) guidelines [43] were used in preparation of the study protocol and manuscript.

### Exposure definition

The Danish National Prescription Registry contains information on sales of prescription drugs, date for sales, specification of the medication, and drug classification codes (the Anatomical Therapeutic Chemical (ATC) classification system, World Health Organization) [40]. Pregnant women with redeemed prescriptions for an SSRI during the period from 30 days before LMP through 91 days after LMP were regarded as exposed during first trimester. Pregnancies without a redeemed prescription in this period were considered unexposed. The following SSRI-preparations were examined (ATC-codes): Fluoxetine (N06AB03), Citalopram (N06AB04), Sertraline (N06AB06), Fluvoxamin (N06AB08), and Escitalopram (N06AB10), both combined and the five latter individually. When specific preparations were examined, pregnant women using multiple preparations of SSRIs were excluded from the analyses.

### Outcome definition

Cases with CHD were identified and verified by trained pediatricians, who have reviewed birth and death certificates, discharge diagnosis from hospitals, post-mortem examinations, and reports from chromosome laboratories for all fetuses and children included in the study. All CHD detected in fetuses or during the first 5 years of life were included. Children who moved out of the County of Funen before the diagnosis of an anomaly, were not included in the Danish EUROCAT Register [33]. Anomalies were coded in ICD9 and ICD10 with the extension from British Paediatric Association and classified according to the EUROCAT classification in subgroups [32]. The group of severe CHD included ICD10-BPA: Q200, Q203, Q204, Q212, Q213, Q220, Q224, Q225, Q226, Q230, Q234, Q251, Q262 and ICD9-BPA: 74500, 74510, 7452, 7453, 7456, 7461, 7462, 74600, 7463, 7467, 7471, 74742. Non-severe CHD included ICD10-BPA: q210, q211, q221, q250 and ICD9-BPA: 7454, 7455, 74601, 7470. We investigated all cases with a CHD without known associated chromosomal anomalies, genetic syndrome or microdeletion. Patent ductus arteriosus in preterm born infants were not included in EUROCAT. ASD were included if still open six months after birth.

### Socioeconomic measures

Information on maternal education from Statistics Denmark was extracted for the year of conception. Highest educational level was used for non-students, while students were classified according to the educational level they would obtain after completion of their education. Maternal education was categorized into three groups: Women with compulsory level education (9 years) were classified as the ‘basic’ category. Women with medium tertiary educations, i.e. bachelors degree education and higher were classified as the ‘higher’ category. The remaining group were classified in the ‘intermediate’ category. Information on maternal income was derived from the year before conception and was categorized in quintiles of the income for all pregnancies each year. Maternal country of origin reflected country of birth. The category “Western” included women from countries in the European Union (EU) except Denmark, and from Eastern Europe, Canada, United States, Australian, and New Zealand. Women from other countries were included in the category “Non-Western”. Data on marital status was extracted as of the year of conception. For unmarried women, the categories “singles” and “cohabiting” were determined by, whether a man with an age difference of less than 15 years who was not a blood relative lived in the same household as the pregnant woman. Women with registered partnerships i.e. same sex marriages were included in the category “married”.

### Statistical analyses

Maternal characteristics were compared with Pearson’s χ^2^-test. The level for statistical significance was defined as a two sided P value less than 0.05. We used Logistic Regression to compute crude and adjusted odds ratios (ORs) with associated 95% confidence intervals (95% CIs) for different subgroups of CHD. Covariates examined included maternal age (≤24, 25–29, 30–34, 35–39, ≥ 40 years), years of conception (1995–1997, 1998–2001, 2002–2004, 2005–2008), and redeemed prescriptions for antiepileptics and/or insulin from 30 days before LMP to 91 days after (yes or no). All data on covariates were extracted from administrative registers and were complete for the study population. In order to detect any additive interaction between maternal socioeconomic status and SSRI-use on risk of congenital anomaly, we used Binomial Regression to calculate adjusted risk difference (RD) with associated 95% CIs.

To examine a possible confounding by indication, i.e. that the underlying disease and not the medication would account for any associations found, the risk of CHD in the offspring of women who had put their treatment of SSRIs on hold during pregnancy, was compared with the risk in the offspring of unexposed women. Women, who used SSRIs from 3–12 months before LMP and within one year after pregnancy, but not in the defined exposure window, were expected having a mental illness without being exposed to SSRIs during pregnancy. Unexposed were in this case defined as women, not treated with SSRIs from one year before to one year after pregnancy. The study population for this sub-analysis consisted of women with LMP between 1st January 1996 and 4th October 2007. Moreover, the association between maternal SSRI exposure and CHD were adjusted for contact with the psychiatric system within one year before pregnancy delivered from The Psychiatric Research Central Register [44]. Finally, we conducted a Change-In-Estimates analysis to examine whether socioeconomic status confounded the association between maternal SSRI-use and CHD. All analyses were conducted in the statistic software STATA (version 12.1).

### Ethics

The study is a register-linkage study of routinely collected data. According to Danish legislation such data linkages have to be approved by the Danish Data Protection Agency. The national and the regional Committees on Health Research Ethics refer to the Danish Data Protection Agency for approval of pure register-linkage studies. The data linkages for this particular project are approved by the Danish data Protection Agency (jr no. 2011-231-0098 and 2010-41-5082).

## Results

### Characteristics of the study population

Among the 72,280 pregnant women who were enrolled in the study, a total of 845 (1.2%) had redeemed at least one SSRI-prescription during the exposure window. The majority had redeemed prescriptions for only one type of SSRI (91.7%), of which Citalopram was most commonly redeemed (35.4%). During the study period from 1995–2008 the proportion of women who used SSRIs increased by eight times. The basic characteristics of the study population are shown in Table 1. SSRI-users differed from non-users in relation to socioeconomic position, as they tended to be less educated, had lower income and were more often singles. We found no statistically significant difference in relation to country of origin. Exposed women had redeemed similar amounts of prescriptions for SSRIs regardless of socioeconomic positions (data not shown). Women taking SSRIs in first trimester were more likely to be in the youngest or oldest age group, smokers, or to be users of antiepileptics and/or insulin compared with non-users (Table 1). SSRI-users and non-users did not differ with respect to plurality, previous miscarriage history, or parity (data not shown).

**Table 1:** **Characteristics of exposed to SSRIs versus unexposed**

Characteristics	Unexposed (n = 71435) n (%)	Exposed to any SSRI (n = 845) n (%)	p value*
**Year of conception**			
1995-1997	16793 (23.51)	54 (6.39)	<0.001
1998-2001	21845 (30.58)	145 (17.16)	
2002-2004	15647 (21.90)	212 (25.09)	
2005-2008	17150 (24.01)	434 (51.36)	
Missing	0 (-)	0 (-)	
**Educational level**			
Basic	12279 (17.19)	251 (29.70)	<0.001
Intermediate	34016 (47.62)	370 (43.79)	
Higher	25140 (35.19)	224 (26.51)	
Missing	0 (-)	0 (-)	
**Income group**			
Low	11715 (16.40)	175 (20.71)	<0.001
Below average	14840 (20.77)	250 (29.59)	
Average	15148 (21.21)	188 (22.25)	
Above average	15100 (21.14)	125 (14.79)	
High	14632 (20.48)	107 (12.66)	
Missing	0 (-)	0 (-)	
**Country of origin**			
Danish	64355 (90.09)	772 (91.36)	0.337
Other Western	1818 (2.54)	22 (2.60)	
Non-Western	5262 (7.37)	51 (6.04)	
Missing	0 (-)	0 (-)	
**Marital status**			
Single	11701 (16.38)	254 (30.06)	<0.001
Cohabiting	30812 (43.13)	328 (38.82)	
Married	28922 (40.49)	263 (31.12)	
Missing	0 (-)	0 (-)	
**Maternal age**			
≤ 24 years	13110 (18.35)	172 (20.36)	<0.001
25-29 years	27506 (38.50)	291 (34.44)	
30-34 years	22303 (31.22)	236 (27.93)	
35-39 years	7507 (10.51)	120 (14.20)	
≥ 40 years	1009 (1.41)	26 (3.08)	
Missing	0 (-)	0 (-)	
**Smoking during pregnancy**			
Yes	13182 (18.45)	271 (32.07)	<0.001
No	51537 (72.15)	511 (60.47)	
Missing	6716 (9.40)	63 (7.46)	
**Use of antiepileptics and/or insulin during pregnancy**			
Yes	782 (1.09)	27 (3.20)	<0.001
No	70653 (98.91)	818 (96.80)	
Missing	0 (-)	0 (-)	

SSRI, selective serotonin-reuptake inhibitor. *χ^2^ test were used to calculate the overall p value for comparison of pregnancies exposed to SSRIs in first trimester with unexposed. Information on smoking during pregnancy and multiple pregnancies were only available for live- and stillbirths.

### Maternal SSRI-use and risk of congenital heart defects

In total, we identified 546 children or fetuses with CHD (519 live births, 12 stillbirths, 2 miscarriages, and 13 termination of pregnancy) and of these were 11 exposed to SSRIs in the first trimester of pregnancy. Table 2 shows that maternal exposure to any SSRI during first trimester was associated with an increased risk of severe CHD of a factor four. The risk estimates for the association between SSRIs and respectively Ebstein’s anomaly (AOR 35.31 (95% CI 3.21-399.43)) and hypoplastic right heart (AOR 8.62 (95% CI 1.01-73.61)) were statistically significant, however, the confidence intervals were very wide. When we studied specific SSRIs, the increased risk of CHD became statistically insignificant. After restriction of the study population to include only live born children, the risk estimates for the association between severe CHD and respectively Citalopram and Escitalopram became statistically significant and the risk estimates for SSRIs overall and severe CHD were increased (data not shown).

**Table 2:** **Risk of congenital heart defects among exposed to SSRIs versus unexposed**

	Unexposed (n = 71435)	Exposed to any SSRI (n = 845)		
	n (%)	n (%)	OR (95% CI)	AOR ^¤^ (95% CI)
Congenital heart defects*	535 (0.75)	11 (1.30)	1.75 (0.96-3.19)	1.64 (0.89-3.00)
**VSD**	329 (17.88)	4 (16.67)	1.02 (0.38-2.76)	0.94 (0.35-2.53)
**ASD**	94 (5.11)	3 (12.50)	2.70 (0.85-8.55)	2.82 (0.88-9.04)
**Pulmonary valve stenosis**	52 (2.83)	2 (8.33)	3.26 (0.79-13.39)	3.47 (0.83-14.50)
**Severe CHD**	123 (0.17)	6 (0.71)	4.15 (1.82-9.44)	4.03 (1.75-9.26)

SSRI, selective serotonin-reuptake inhibitor. CHD, congenital heart defects. VSD, ventricular septal defects. ASD, atrial septal defects. NE, no estimate. OR, odds ratio. AOR, adjusted odds ratio.

*Congenital heart defects include both non-severe and severe CHD.

Estimates are presented as ORs with 95% confidence intervals (95% CIs).

^¤^Multivariable Logistic Regressions are adjusted for maternal age, year of conception, use of antiepileptics and/or insulin during first trimester.

Table 3 presents the adjusted risk differences for severe CHD between exposed and unexposed women stratified according to socioeconomic groups. Formal tests for interaction did not reach statistical significance, however, the risk differences may suggest a higher risk of severe CHD in the offspring of women exposed to SSRIs who were cohabiting, had a basic education, and high income.

**Table 3:** **Risk difference of severe congenital heart defects between exposed and unexposed stratified by socioeconomic groups**

	Severe CHD (n = 129)	
	n (%)	ARD ^¤^ per 1,000 exposed to SSRIs (95% CI)
**Marital status**		
Single	25 (19.38)	1.17 (-5.71; 8.06)
Cohabiting	52 (40.31)	7.19 (-3.00; 17.37)
Married	52 (40.31)	5.71 (-4.66; 16.07)
**Maternal educational level**		
Basic	40 (31.01)	8.42 (-4.79; 21.63)
Intermediate	53 (41.09)	4.00 (-3.49; 11.49)
Higher	36 (27.91)	3.21 (-5.76; 12.18)
**Maternal income group**		
Low	24 (18.60)	2.90 (-6.83; 12.63)
Below average + average	55 (42.64)	4.53 (-2.93; 12.00)
Above average + high	50 (38.76)	6.80 (-4.96; 18.56)

SSRI, selective serotonin-reuptake inhibitor. CHD, congenital heart defects. ARD, adjusted risk difference. Estimates are presented as ARD with 95% confidence intervals (95% CIs).

^¤^Multivariable Binomial Regressions are adjusted for maternal age, year of conception, and use of antiepileptics and/or insulin during first trimester. Categories of maternal income group are combined in order to ensure, that all categories contain exposed cases. No exposed cases had other origin than Danish, why estimates could not be stratified in relation to country of origin.

### Assessment of biases

Inclusion of maternal socioeconomic variables as covariates in the multiple Logistic Regression-model resulted in a minor (less than 10%) change of the estimated risk of CHD with maternal exposure to SSRIs. In order to assess a possible confounding by indication, the association between maternal SSRI exposure and CHD were adjusted for maternal contact with the psychiatric system within one year before pregnancy. This adjustment did not change the risk estimates (data not shown). Importantly, we found that the offspring of women who used SSRIs from 3–12 months before LMP and within one year after pregnancy (4 cases), but not in the defined exposure window, had an increased prevalence of CHD compared with the offspring of unexposed (not using SSRIs from one year before to one year after pregnancy) (AOR 3.34 (95% CI 1.59-11.81)).

To assess the implications of misclassification, we changed the exposure window in sub-analyses: Including women who redeemed prescriptions during the period from three months before to three months after LMP marginally reduced the risk estimates, while inclusion of redeemed prescriptions only during first trimester did not affect the estimates. Exclusion of women who redeemed prescriptions for SSRIs 2–3 months before LMP and in the second and third trimesters, but not in the defined exposure window, i.e. with a high risk of being misclassified as exposed, did not changes in the risk estimates.

## Discussion

### Main findings

In this cohort study using high quality anomaly data from the Danish EUROCAT Register we found use of any SSRI in the first trimester to be associated with increased risk of severe CHD. The study lacked statistical power to document an interaction between socioeconomic status and maternal SSRI-use on the risk of severe CHD.

### Accordance with other studies

Three previous Danish studies have found that CHD are more prevalent in children of pregnant women who redeemed prescriptions for SSRIs during first trimester of pregnancy [16, 18, 22]. Administrative register data on live births from The Danish National Hospital Register formed the basis for the outcome data in these three studies. The recent Danish nationwide cohort study of Jimenez-Solem et al. [16] included 848,786 pregnancies between 1997–2009. Among these pregnancies, 4,183 (0.5%) were exposed to SSRI during first trimester. Maternal exposure to any SSRI were found to be associated with a two-fold increase in the risk of CHD overall (AOR 2.01 (95% CI 1.60-2.53)). Several studies from other countries have also suggested an association between maternal SSRI-use and CHD, but the results regarding specific types of SSRIs are conflicting [12–23].

In our study, the overall risk estimate for CHD was close to unity and not statistically significant, but for severe CHD the risk estimate was high and statistically significant (4.03 (1.75-9.26)). This could indicate that SSRIs are involved in the etiology of severe CHD only. Our findings suggest an increased risk of Ebstein’s anomaly and hypoplastic right heart after maternal exposure to SSRIs although numbers were very small (one case with Ebstein and one with hypoplastic right heart). Two previous studies, which have examined specific diagnoses of CHD, have found an increased risk of right ventricular outflow tract obstruction defects (including Tetralogy of Fallot, transposition of great arteries, pulmonary atresia) for paroxetine [12, 19, 20]. We could not confirm the increased risk for ASD and VSD found in studies using administrative databases [16, 22]. We identified 7 different diagnoses in the 6 exposed cases with severe CHD and this lack of specificity should lead to cautiousness.

According to our knowledge no other published studies have examined the association between SSRI-use and congenital anomalies stratified by socioeconomic groups. Unfortunately, this data material did not provide sufficient statistical power to determine such an interaction, since both the exposure and the outcome are rare events. The estimates show opposite trends regarding the risk of severe CHD in exposed with different socioeconomic positions, where different measures of socioeconomic position were used. It is likely that these observations are due to the great uncertainty associated with the estimates.

### Strengths and limitations

The main strength of the study is the high quality of data on congenital anomalies from the Danish EUROCAT Register including also the most severe congenital anomalies leading to fetal death and terminations of pregnancy. The verification of anomalies diagnoses in EUROCAT results in low risk of misclassification of the outcome variable and a high case ascertainment. In contrast, the standard reporting of congenital anomalies to administrative databases is associated with great risk of errors in diagnosis coded and is often limited to diagnoses of live born children. In particular, these databases have a tendency to overestimate the prevalence of septum defects [45]. The downside of using data from the Danish EUROCAT Register is the limited size of the study population, which has consequences in relation to lack of statistical power. We examined different diagnoses of CHD pooled in a group in order to provide sufficient statistical power. However, grouping CHD diagnoses with heterogeneous etiology will result in a dilution of the studied association. When we limited the study population to include only live births, the size of the risk estimate for severe CHD was increased, and the association between severe CHD and Citalopram and Escitalopram respectively, became statistically significant. This indicates that CHD are underdiagnosed in fetal deaths and abortions [33, 46].

Although the Register of The Danish National Prescription Registry includes approximately 97.5% of all redeemed prescriptions [47] the risk of misclassification of the exposure was significant. We have no knowledge of when and whether the pregnant women used the medication they had redeemed prescriptions for. Furthermore, we have no information about medication given during hospitalization. In order to verify whether the women actually took the medication, we would have to ask them, which was not possible in the present study. Misclassification of the exposure variable will probably lead to bias towards an underestimation of SSRI’s effect. The strength, by using prescription data rather than self-reported information about SSRI-use, is that recall bias and selection bias are eliminated. The definition of the exposure window was based on the average number of tablets in a package with SSRIs. Results from a sub-analysis indicated that a broader definition of the exposure variable would lead to more non-exposed pregnancies being misclassified.

Information on covariates was obtained from Danish administrative registers, which in general are considered to be of high quality, limiting the extent of bias [48]. However, these registers are not designed to be used for epidemiological studies, which is why information on important potential confounders was not available. We studied confounding by indication by adjusting for contact with the psychiatric system. The measure’s validity is low in relation to reflect maternal mental illness, and the adjustment did not lead to changed risk estimates. In accordance with findings of Jimenez-Solem et al. [16], we found an association towards an increased risk of CHD in the offspring of women who had used SSRIs until 3–12 months before the pregnancy and within one year after the pregnancy, but not in the exposure window. Jimenez-Solem et al. [16] assumed that these women probably have a mental illness without being exposed during pregnancy, and the increased prevalence of CHD indicates the presence of confounding by indication. However, it is likely that women who have experienced having a malformed child are more inclined to resume their use of SSRIs after pregnancy, why the finding instead may be explained by differential misclassification. Published studies which have used a comparison group of mentally ill women treated with other antidepressants than SSRIs, find a correlation between SSRI-use during early pregnancy and congenital anomalies [14, 25]. We are therefore less inclined to believe that confounding by indication plays a major part in the association.

We adjusted for years of pregnancy, because use of SSRIs among pregnant women have been increasing during the study period [5], and there are an increased amount of anomaly diagnosis [49]. We classified students according to the educational level they will achieve, when they have completed their education, because we believe, that most students share lifestyle behaviour and attitudes with their future colleagues. Information on income for the year prior to conception was used in order to prevent the pregnancy itself affecting the used measure for income. It can be discussed whether income is a valid measure of socioeconomic status, as it classifies students who are taking a higher education, in the lowest socioeconomic group. We distinguished between Danish women, women from other Western countries, and women from non-Western countries, because we assumed that they differ in relation to lifestyle and health behaviour. However, this division is very rough and heterogeneous ethnic groups are combined, which may have consequences in terms of residual confounding. Users of antidepressants tend to have a lower socioeconomic status compared with non-users, and previous studies have shown an increased prevalence of congenital anomalies [50–52], including CHD in the offspring of women with low socioeconomic position [34–36]. We therefore examined the role of maternal socioeconomic status as a confounder for the association between SSRI-use and CHD in a sub-analysis. However, our finding did not suggest that maternal socioeconomic status confounds the association.

### Public health perspective of SSRI-use during pregnancy

The study’s results with respect of an association between SSRI-use and severe CHD are generalizable, but the influence of socioeconomic status is more contextual. Although statistically significant, the increased risks associated with SSRI-exposure are small in absolute terms. We estimated that annually 4.19 severe CHD can be prevented in the 65,439 pregnancies (which minimum amounts to 12th gestation week in Denmark) by eliminating exposure to SSRIs during first trimester. However, use of SSRIs during pregnancy has been associated with other adverse pregnancy outcomes e.g. miscarriages, preterm births, low birth weight and persistent pulmonary hypertension [29, 53–55], and we do not know the long term consequences of exposure to SSRIs in utero. For each pregnancy potential consequences of SSRI-use should be weighed against the impact on the child and the mother of an untreated mental disorder.

## Conclusion

The study was not able to document whether the risk of CHD after maternal use of SSRIs differ in various socioeconomic positions. In this study, using an optimised case ascertainment compared to previous studies, we found an increased risk of severe CHD with maternal use of SSRIs during first trimester. The present study did not support findings from previous studies where information on congenital anomalies was based on administrative data, in which an association between SSRI and septal defects is observed.

## Authors’ information

EG has been responsible for the Danish EUROCAT Registry since 1988.

## Abbreviations

SSRI: Selective serotonin-reuptake inhibitors
AOR: Adjusted odds ratios
ARD: Adjusted risk difference
LMP: Last menstrual period
GA: Gestational age
95% CI: Associated 95% confidence interval.
